# The role of short-chain fatty acids (SCFAs) in regulating stress responses, eating behavior, and nutritional state in anorexia nervosa: protocol for a randomized controlled trial

**DOI:** 10.1186/s40337-023-00917-6

**Published:** 2023-10-26

**Authors:** Robin Quagebeur, Boushra Dalile, Jeroen Raes, Lukas Van Oudenhove, Kristin Verbeke, Elske Vrieze

**Affiliations:** 1 Mind-Body Research, Department of Neurosciences, KU Leuven, Leuven, Belgium; 2 Leuven Brain Institute, KU Leuven, Leuven, Belgium; 3 Translational Research Center for Gastrointestinal Disorders (TARGID), Department of Chronic Diseases and Metabolism, KU Leuven, Leuven, Belgium; 4 Laboratory of Molecular Bacteriology, Department of Microbiology and Immunology, Rega Institute, KU Leuven, Leuven, Belgium; 5 Center for Microbiology, Vlaams Instituut Voor Biotechnologie (VIB), Leuven, Belgium; 6 Cognitive and Affective Neuroscience Lab (CANlab), Department of Psychological and Brain Sciences, Dartmouth College, Hanover, NH USA

**Keywords:** Anorexia nervosa, Short-chain fatty acids (SCFAs), Gut-brain axis, Gut microbiome, Eating behavior, Food choice, Stress response, Trier Social Stress Test (TSST), Cortisol, Randomized controlled trial

## Abstract

**Objective:**

This protocol proposes investigating the effects of short-chain fatty acids (SCFAs)—namely acetate, propionate, and butyrate—as mediators of microbiota-gut-brain interactions on the acute stress response, eating behavior, and nutritional state in malnourished patients with anorexia nervosa (AN). SCFAs are produced by bacterial fermentation of dietary fiber in the gut and have recently been proposed as crucial mediators of the gut microbiota's effects on the host. Emerging evidence suggests that SCFAs impact human psychobiology through endocrine, neural, and immune pathways and may regulate stress responses and eating behavior.

**Method:**

We will conduct a randomized, triple-blind, placebo-controlled trial in 92 patients with AN. Patients will receive either a placebo or a mixture of SCFAs (acetate propionate, butyrate) using pH-dependent colon-delivery capsules for six weeks. This clinical trial is an add-on to the standard inpatient psychotherapeutic program focusing on nutritional rehabilitation.

**Hypotheses:**

We hypothesize that colonic SCFAs delivery will modulate neuroendocrine, cardiovascular, and subjective responses to an acute laboratory psychosocial stress task. As secondary outcome measures, we will assess alterations in restrictive eating behavior and nutritional status, as reflected by changes in body mass index. Additionally, we will explore changes in microbiota composition, gastrointestinal symptoms, eating disorder psychopathology, and related comorbidities.

**Discussion:**

The findings of this study would enhance our understanding of how gut microbiota-affiliated metabolites, particularly SCFAs, impact the stress response and eating behavior of individuals with AN. It has the potential to provide essential insights into the complex interplay between the gut, stress system, and eating behavior and facilitate new therapeutic targets for stress-related psychiatric disorders.

*This protocol is prospectively registered with ClinicalTrials.gov, with trial registration number NCT06064201.*

## Introduction

### Background

As a potent modulator of the bidirectional communication between the host's gastrointestinal tract and brain, the gut microbiota plays a central role in what's commonly known as the gut-brain axis. The gut microbiota can impact multiple bodily processes such as metabolism, immunological responses, body weight regulation, brain function, and eating behavior [1–3]. Concurrently, diet greatly influences the gut microbiota composition [4], suggesting a complex interplay. In particular, dietary fiber intake has been identified as a primary factor determining gut microbiota composition and function. Dietary fiber is considered an important substrate that helps to preserve gut ecology and regulate host physiology [5]. In patients with anorexia nervosa (AN), who characteristically maintain highly restricted diets, significant alterations in gut microbiota composition have been reported [6–9]. Moreover, shifts in the gut microbiota composition of patients with AN have been observed before and after renourishment. However, even after renourishment, the gut microbiota composition continues to differ from healthy controls [10–14]. Changes in gut microbiota composition have also been considered a potential contributing factor to gastrointestinal symptoms, such as constipation and bloating, which are frequently experienced by this patient population [15]. Current literature on nutritional rehabilitation in AN recommends energy-rich diets. Further, it proposes that these diets should be high in both fat and fiber to help establish a gut microbiome that more closely mirrors healthy individuals [16].

Short-chain fatty acids (SCFAs), metabolites produced following the bacterial fermentation of dietary fiber in the large intestine, are considered candidate mediators of the gut bacteria's effects on the host [17]. Acetate, propionate, and butyrate are the most abundant SCFAs and may affect the host through immune, endocrine, neural, and humoral pathways [18]. Stress is the physiological response to a challenging physical, emotional, or cognitive encounter that exceeds the body's coping resources and activates the sympatho-adrenal medullary pathway (SAM) and the hypothalamus–pituitary–adrenal (HPA) axis. Interestingly, recent evidence suggests that SCFAs mediate the effects of gut bacteria on stress responses of the HPA axis [19–21]. Oral supplementation of SCFAs in rodents reduced corticosteroid response to acute stress, reduced stress-induced gastrointestinal permeability, and reversed changes in anhedonia [20, 21]. Human translational research found that administering SCFAs to the large intestine attenuated the cortisol response to acute psychosocial stress [19]. While the stress response can be adaptive and beneficial, prolonged or exaggerated stress can heighten the risk of developing neuropsychiatric disorders [22]. The involvement of the HPA axis in individuals with AN has been implicated in the development and perpetuation of this disorder [23–26]. Researchers hypothesize that eating disorder-related behaviors, such as restricting, binging, purging, or excessive physical exercise, may function as dysfunctional adaptations to alleviate and manage negative affective states [27–29]. These maladaptive eating behaviors often increase in response to stress and negative emotions [30, 31]. This consequently forms a vicious cycle, where the continued physiological state of nutritional deprivation can lead to subjective and objective stress activation (e.g., via chronic HPA axis stimulation and autonomic nervous system alterations) [23, 32, 33]. The possible role that the gut microbiota and SCFAs might play in the maladaptive stress responses and related eating behaviors characteristic of AN is intriguing and presents a promising research avenue to expand our understanding of this complex disorder.

Data on SCFAs in AN is limited, but some studies have reported lower fecal concentrations of SCFAs [6, 7, 13, 34, 35]. One study examined fecal SCFAs in patients with AN and observed their nutritional intake. They found that individuals with AN had lower fecal concentrations of butyrate and propionate, a normal protein and fiber intake, and lower carbohydrate and fat intake [35]. A systematic review of microbiological data suggests that characteristic alterations in the gut bacteria of patients with AN include a decrease in butyrate-producing species and an increase in species that degrade mucin, which increases gastrointestinal permeability [9]. However, current findings on fecal short-chain fatty acid (SCFAs) concentrations in AN are inconclusive, as fecal SCFAs only provide information on non-absorbed SCFAs and do not reflect in situ production rates, absorption, or interactions with other biologically relevant molecules or cell types. A cross-sectional study on serum SCFAs found significantly lower levels of butyric, isobutyric, and isovaleric acid in individuals with active and recovered AN [36]. Despite a growing body of evidence indicating a relationship between the gut microbiota and AN, there is a significant gap in research that causally examines the role of SCFAs and explores the interactions between the gut microbiota, SCFAs, stress response, and eating behavior in AN.

### Study aims

Building on the current gaps in the literature, we designed a triple-blind, randomized, placebo-controlled trial to examine the role of SCFAs as mediators of microbiota-gut-brain interactions in AN. Specifically, we aim to assess the effects of SCFAs on the acute stress response, eating behavior, and nutritional status in patients with AN following SCFA delivery to the large intestine using pH-dependent colon-delivery capsules (CDCs). This method allows the administration of SCFAs in exact amounts in a chronically tolerable manner while mimicking, as closely as possible, the process of bacterial fermentation of dietary fiber in the gut. We hypothesize that colonic SCFA administration daily for six weeks will modulate neuroendocrine [cortisol, adrenocorticotropin hormone (ACTH), salivary-alpha amylase], cardiovascular (heart rate, heart rate variability), and emotional responses (subjective stress, anxiety, perceived control, hunger and desire to eat) to acute laboratory stress. Additionally, we predict that the intervention will result in a shift in dietary choices towards less restricted choices, as captured during a food choice task (FCT), and an improvement in the nutritional state, reflected by a greater increase in BMI.

The longitudinal design of this study provides a unique opportunity to examine the causal role of SCFAs in stress and eating behaviors in individuals with AN. By stepping away from the typical cross-sectional studies in the gut microbiota research field, our work can yield new insights into the complex interplay between the gut, stress system, and eating behavior and facilitate new therapeutic targets for stress-related psychiatric disorders.

## Methods

The Standard Protocol Items: Recommendations for Interventional Trials (SPIRIT) checklist [37] was used as a guideline for this protocol.

### Study design and setting

This study is a monocentric, randomized, triple-blind, placebo-controlled trial with two parallel arms. Participants in both groups will receive concomitant treatment as usual, consisting of an inpatient psychotherapeutic program of 12 weeks focusing on nutritional rehabilitation. Using a block randomization method, we will randomly assign participants to the placebo or SCFA group with a 1:1 allocation ratio. The total duration of the study is 12 weeks, including a pre-intervention baseline visit, which takes place within the first week of hospitalization, a post-intervention visit at six weeks, and a follow-up visit at 12 weeks. The study will be conducted at the Leuven University Psychiatric Hospital (UPC Z.Org), Belgium.

### Sample size

Based on our previous work [19], we calculated, using the General Linear Multivariate Model Power and Sample Size software (GLIMMPSE), that a total sample size of 70 participants (n = 35 per treatment arm) yields sufficient power (80%) to detect the previously found effect of SCFAs on the cortisol response to an acute stress paradigm. Given that no information is available to calculate the effects of SCFAs on stress-induced cortisol response in patients with AN where greater variability might be present, we increased the sample size to 80 participants (n = 40 per arm). Based on a sensitivity power calculation (G*Power 3.1.9.2 software), 40 participants per arm would enable us to achieve 80% power to detect a small effect (Cohen's f = 0.16) in a within-between 2 (“visit”, baseline vs. post-intervention) × 2 (“treatment”, placebo vs. SCFA) mixed ANOVA model. Taking into consideration a 15% risk of dropout, we aim to recruit 92 individuals in total.

### Eligibility criteria

Females of at least 16 years of age, diagnosed with AN per DSM-5 guidelines—having first met these DSM-5 criteria less than seven years ago—and having a current Body Mass Index (BMI) of less than 17.5 kg/m^2^ will be recruited. Further, participants must be Dutch-speaking and agree to participate in the inpatient therapeutic program on the specialized eating disorder unit of the hospital. Regarding the exclusion criteria, we consider several factors that potentially impact the stress task used in the study. These include substance or alcohol abuse, chronic use of sedatives or sleep medication, and a high caffeine intake, defined as more than 1000 ml of coffee daily or equivalent quantities of other caffeine-containing substances. Moreover, we will exclude individuals with medical or psychiatric conditions that may interfere with study procedures or risk patient well-being. Medical criteria for exclusion encompass gastrointestinal issues like Crohn's disease and ulcerative colitis, as well as individuals who are medically unstable due to severe malnutrition. Regarding psychiatric exclusions, individuals experiencing acute suicidal thoughts or behaviors are not eligible to participate. Additional exclusion factors relate to their potential impact on the microbiota composition. These include recent use of pre- or probiotics (within the last month before the study), use of antibiotics (within the previous three months before the study), and use of antipsychotics. However, other drugs will be permitted if the patients have been on a stable dosage for at least four weeks.

### Recruitment

The research team will contact potential participants diagnosed with AN and on the waiting list for admission to the eating disorder unit at UPC Z.Org to schedule an online appointment to receive detailed information about the study and have any questions answered. Subsequently, participants will receive a comprehensive informed consent form outlining the study protocol. Finally, upon hospital admission, the investigator will conduct a final eligibility assessment, and participants (or those under 18, along with parental consent) will provide written consent or assent.

### Randomization and blinding

This study will use computer-generated block randomization with random block sizes of 2 and 4 for participant allocation. There will be no stratification applied during the randomization process. An independent researcher not involved in any aspect of the trial will securely store the allocation sequence. The study will be performed in a triple-blinded manner, such that the researchers, patients, and data analysts remain unaware of treatment allocation until final statistical results are obtained.

### Interventions and concomitant care

We will efficiently deliver SCFAs to the large intestine by utilizing pH-dependent CDCs to mimic the process of dietary fiber fermentation in the colon, as done previously [19]. Participants will consume capsules of a mixture of SCFAs spread at four intervals throughout the day. The SCFA mixture contains acetate, propionate, and butyrate in a ratio of 60:20:20, and the total daily amount of SCFAs is equivalent to the fermentation of 10 g of arabinoxylan oligosaccharides [38]. Microcrystalline cellulose, not absorbed in the gastrointestinal tract, will be included in the same type of CDCs to serve as a placebo. The nursing staff will distribute the capsules to the participants individually, supervise their intake, and monitor adherence by capsule count. Although adverse effects are not expected [19], these will be documented. Participation will be discontinued if the patient exhibits a high medical or psychiatric risk requiring priority or fails to adhere to the consumption of the intervention substances (taking less than 80% of capsules) or to the study procedures.

Participants in both groups will receive treatment as usual, consisting of an inpatient psychotherapeutic program of 12 weeks focusing on nutritional rehabilitation. We will closely monitor the use of other medications throughout the study. However, such usage will not lead to the termination of the trial intervention. After undergoing the intervention and post-intervention visit, if any participant decides to leave the residential program early, we will still aim for a follow-up visit to take place at the hospital.

### Study visits

#### Baseline and post-intervention visit

Participants will be asked to collect a stool sample during the first three days of hospitalization—before the start of the intervention—and the three days preceding their second study visit. We will instruct them to refrain from vigorous physical activity, alcohol, and caffeine one day before the visits to prevent interference with the scheduled tasks. To standardize fiber intake, the participants will receive a low-fiber standardized dinner the night before each visit and a low-fiber breakfast and lunch on the day of the visits. Low-fiber snacks on both visits will replace the snacks provided on the ward at 10:30 and 15:00. Participants will keep an electronic food diary (Nutritics software) for three days during their initial hospitalization and three days before their second study visit to monitor their macronutrient and energy intake. To avoid encouraging disordered eating behaviors, the diary’s daily energy intake, and other nutritional reports will not be accessible to participants.

**Fig. 1:**
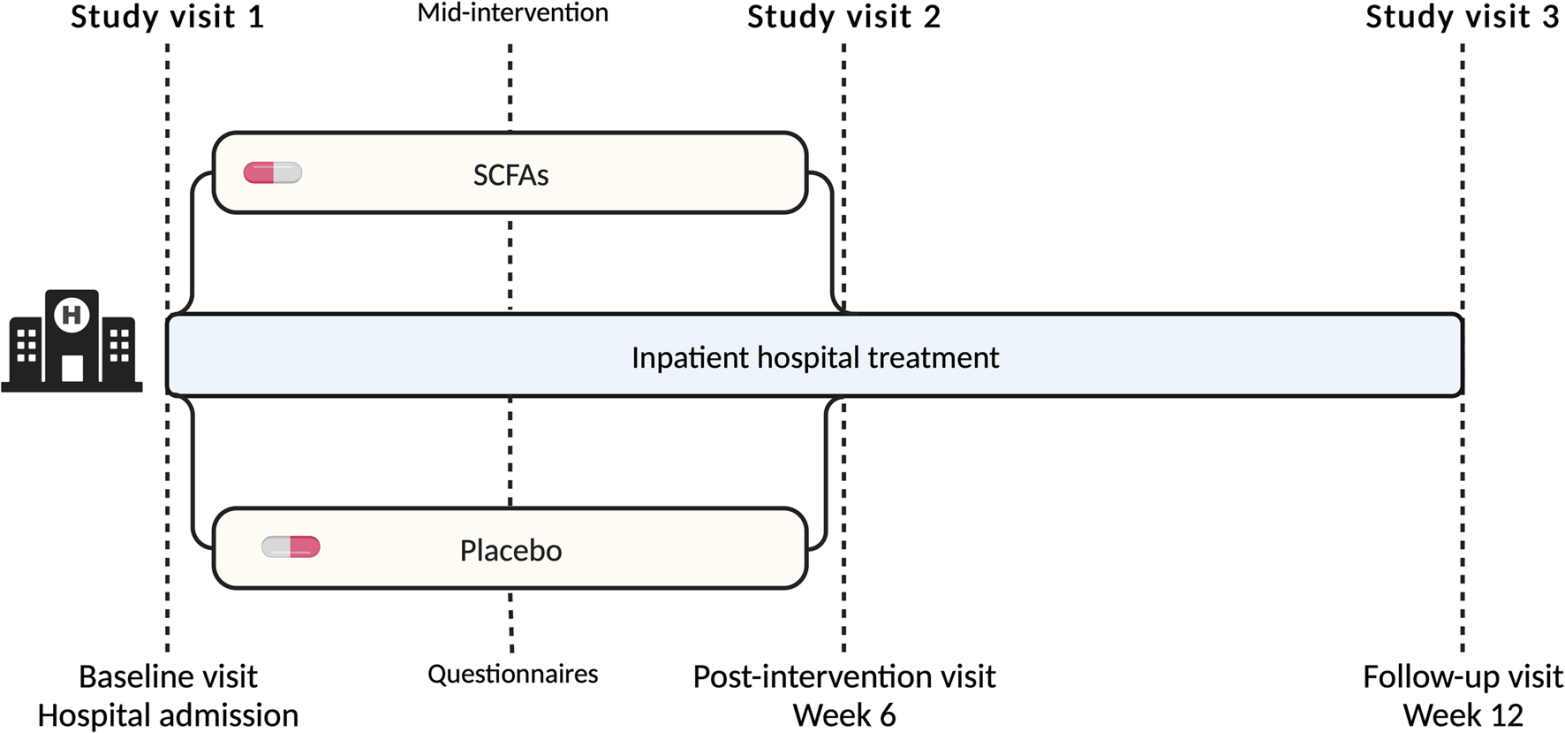
Visits overview

**Fig. 2:**
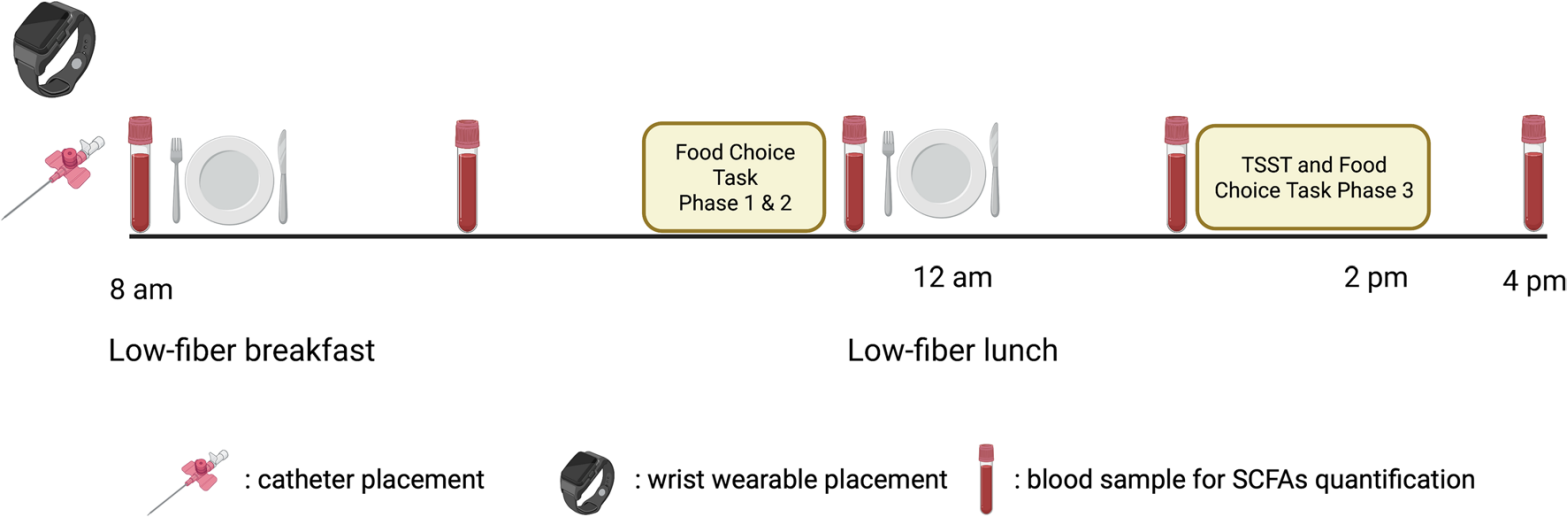
Baseline and post-intervention visit

We will insert an intravenous (IV) catheter at the start of each visit to collect multiple blood samples throughout the day (Figs. 1, 2). To avoid interference with cortisol analysis during a later stress task, we will place the catheter in the morning [39]. Along with the catheter insertion, the researcher will secure the wearable wrist device onto the participant's non-dominant wrist. The device will be worn continuously for the entire day until the following morning. In the morning, we will ask participants to fill in the battery of self-reported questionnaires detailed in the “Outcomes” section below. At around 11:30, before lunch, participants will complete the first and second phase of the food choice task (FCT). At 13:30, participants will be escorted to the acclimatization room to rest before the Trier Social Stress Test (TSST). The “Procedures” section below provides detailed descriptions of both the FCT and TSST, including their structure and implementation. The stress induction phase of the TSST will begin at 14:00 and will be immediately followed by the computerized third phase—the choice phase—of the FCT. After phase three of the FCT, we will debrief the participants and serve a snack-sized portion chosen by the computer task. The visit concludes with collecting the final blood sample, at which point the IV catheter will be removed.

#### Follow-up visit

Participants will be required to maintain a comprehensive food diary and collect one stool sample during the three days leading up to the visit. During the follow-up visit, the participant’s body weight will be measured again and they will fill in the self-reported questionnaires.

### Procedures

#### Food choice task (FCT)

We will use a FCT developed by Steinglass and colleagues to capture the participants' eating behavior [40]. The computerized task assesses maladaptive restrictive eating behavior in patients with AN, who characteristically choose low-calorie and low-fat foods. In a previous study, the task demonstrated high test–retest reliability in healthy controls [41]. Additionally, researchers validated the task in patients with AN by correlating it with their actual food consumption during a meal the following day (r = 0.61) [42, 43].

The task consists of three phases. During the first two phases, participants will view food images and rate their healthiness and taste using a five-point Likert scale, where “Healthy” to “Unhealthy” represents the health ratings, and “Good” to “Bad” represents the taste ratings. The computer will then assign a reference food item for each participant based on their input, selecting an item they rated as neutral (a score of three) for both taste and healthiness at baseline. In phase three, after undergoing the stress induction of the TSST, they will be asked to make a series of choices between their designated reference food item and other food options. The reference item is presented alongside the other food items on each trial, with the reference item displayed on the left side of the screen. Participants must indicate which item they chose to eat. They can express their preference on a Likert scale, with "strongly prefer" anchored to the left for the reference item and to the right for the other item. This phase of the task aims to capture food choices during stress. Participants are given four seconds per trial to respond during each of the three phases of the FCT. To stimulate authentic food choices, participants are informed that a snack-sized portion from one of their selected items will be served as a snack after the task. The images of the food items used in the task reflect a range of dietary choices typically found in the environment. Half of the options are high in fat, while the other half are low, defined as having less than 30% of total calories from fat. The order of the rating phases and choice trials will be counterbalanced and randomized across participants.

#### Trier social stress test (TSST)

During the baseline and post-intervention visits, participants will complete the TSST, a widely used standardized psychophysiological stress paradigm. The TSST specifically targets social-evaluative threat and perceived control, initiating a robust acute stress response that activates the sympathetic nervous system and adrenocortical stress response [44]. No significant habituation or sensitization has been found when there is a large enough interval between sequential TSSTs within a subject [45, 46]. Slight modifications are made to the speech and arithmetic part of the task to make it less predictable during the second administration.

The TSST consists of three distinct phases: an acclimatization phase, a stress induction phase, and a recovery period. The 20-min acclimatization period stabilizes the participant's physiological responses before starting stress induction. Once this period has elapsed, participants are escorted to a second room to start a five-minute task introduction. When the participant enters the second room, they encounter a two-member jury panel. The lead researcher introduces the task by reading a standardized script aloud, which informs participants that they will be required to deliver a speech for a job application in front of a panel of judges. They are told that they will have five minutes to prepare for the speech and another five minutes to deliver it and that their performance will be video recorded. Following the task introduction, participants are escorted back to the first room to begin the anticipatory stress phase, where they prepare for their mock job interview. Once this period has elapsed, the lead researcher returns participants to the second room and starts the video recording. After the researcher leaves the room, the active speech portion of the task begins, and the jury panel gives participants five minutes to deliver their speech. Upon finishing the speech, one of the panel members introduces the participant to the five-minute surprise arithmetic task for the remaining five minutes of the stress induction phase. Participants are instructed to perform a serial subtraction task where they subtract 13 from 1022 repeatedly (or 17 from 2043 during the second visit), aiming to complete as many subtractions as possible with speed and accuracy. If participants make errors, the panel members will ask them to start over from the beginning. Once the arithmetic task is complete, the lead researcher will return to the room and allow the participant to proceed to the third phase of the FCT. At the end of this task, the lead researcher and panel members will debrief the participant.

## Outcomes

### Primary and secondary outcomes

The primary outcome of this study focuses on the salivary cortisol response to acute laboratory stress. We will examine the change in cortisol levels within and between SCFAs and placebo groups. Crucial secondary outcomes encompass the participants' food choices and nutritional status (BMI). All other measures delineated in the subsequent sections serve as secondary outcome measures. See Table 1 for an overview of scheduled assessments.

**Table 1:**
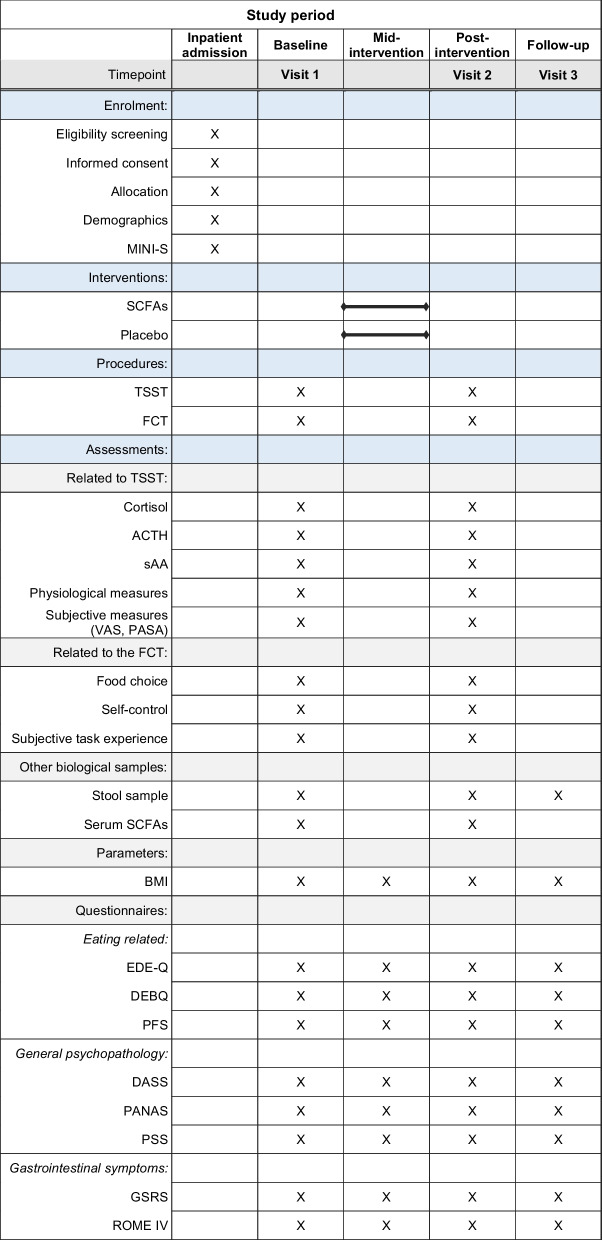
Schedule of enrolment, interventions, and assessments (SPIRIT figure)

### Measurements related to the TSST

#### Neuroendocrine measures

**Fig. 3:**
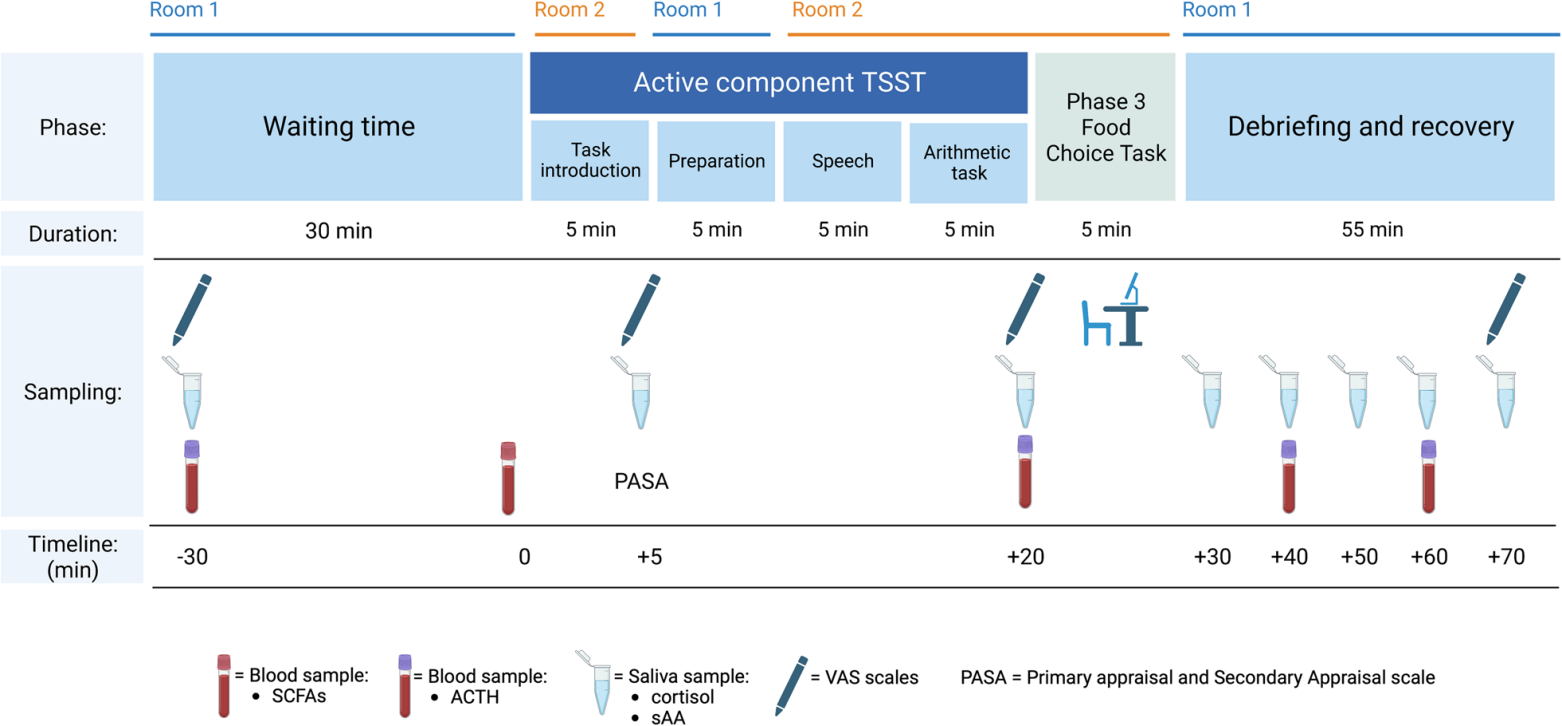
TSST and phase 3 of FCT

To measure the adrenocortical stress response during the TSST, we will collect eight saliva and four blood samples at regular intervals before, during, and after stress induction (see Fig. 3). Saliva samples will be obtained using synthetic Salivette devices and will be used to determine salivary cortisol and alpha-amylase (sAA) levels. Blood samples will be collected through an IV catheter in EDTA tubes to measure ACTH levels. After collection, all samples will be centrifuged and stored at − 80 °C until analysis by ELISA. Additionally, the participants' menstrual cycle phase and any use of hormonal contraceptives will be noted for its effect on the salivary cortisol response to acute stress.

#### Subjective measures

To assess participants' subjective experience during the TSST, we will use Visual Analog Scales (VAS) to measure subjective stress, anxiety, perceived control, hunger, and desire to eat. We will also use the Primary Appraisal and Secondary Appraisal scale (PASA) [47], which assesses four cognitive appraisal processes of stress: threat, challenge, self-concept of own abilities, and control expectancy. Participants will complete this scale after the TSST introduction and before starting the preparation phase.

#### Physiological measures

We will monitor participants' autonomic nervous system activity during both study visits using a wrist device (Empatica EmbracePlus) that tracks heart rate response, heart rate variability, temperature, accelerometry data, and electrodermal activity. Participants will wear the device for the entire day, from the morning of the study visit until the following morning.

### Measurements related to the FCT

The FCT captures the participant's taste, health, and choice ratings on a 5-point Likert scale and their response times. The food choices made during the third phase of the FCT using the 5-point scale are converted into binary responses (yes or no preference for the 'reference item' versus the trial-unique food item). The primary outcome for the FCT is the proportion of high-fat trial-unique food items chosen over the reference item. Additionally, the proportion of self-controlled choices, choosing healthy, less tasty foods or not choosing unhealthy, tasty foods, will be evaluated as a secondary outcome.

Our objective is to measure food choices made under stress by conducting the third phase of the FCT following stress induction. After completing the FCT, participants will complete a self-developed self-report questionnaire on their subjective task experience.

### Biological samples

Blood samples for SCFA analysis will be collected via an intravenous catheter into red top serum glass tubes at five timepoints (8 am, 10 am, 12 am, 2 pm, and 4 pm) during study baseline and post-intervention study visits. The samples will be centrifuged, and the serum will be aliquoted and stored at − 80 °C for later analysis using gas chromatography-mass spectrometry. The stool samples will be used to analyze microbiota composition and quantify fecal SCFA concentrations. Stool samples are collected using in-house developed sampling devices, in which fecal matter is aliquoted during collection in wells containing 0.2 g fecal matter. These samples are sealed and immediately site-frozen at − 20 °C, and transported in a cooler within 48 h to a − 80 °C storage freezer.

### Other measures and questionnaires

During the hospital admission's final eligibility assessment, we will conduct the M.I.N.I-S International Neuropsychiatric Interview [48] to identify potential comorbid psychiatric disorders. Additionally, a self-developed questionnaire will gather information on demographic and clinical parameters. We will also maintain a comprehensive and detailed record of all medications, including antibiotics and any supplements, taken before and throughout the study.

The nursing staff at the hospital unit recurrently measures the participant's weight, while their height is measured during the initial visit. From these data points, we will systematically calculate BMI. In addition to monitoring their autonomic nervous system activity during the TSST, the wearable device will also track their physical activity during both study visits.

Participants will complete self-reported questionnaires on four occasions during the study: on the first, second, and third study visits and three weeks into the intervention between the first and second visits. Changes in eating disorder symptoms will be evaluated using the Eating Disorder Examination Questionnaire (EDE-Q) [49]. Additionally, restrained, emotional, and external eating behavior will be assessed by the Dutch Eating Behavior Questionnaire (DEBQ) [50]. 'Hedonic hunger' is measured through the Power of Food Scale (PFS) [51]. Furthermore, depressive symptoms, anxiety symptoms, affect, and stress will be assessed using the Depression, Anxiety, Stress Scales (DASS) [52], the Positive and Negative Affect Schedule (PANAS) [53], and the Perceived Stress Scale (PSS) [54]. Gastrointestinal complaints are scored using the Gastrointestinal Symptom Rating Scale (GSRS) [55] and the Rome IV questionnaire for functional gastrointestinal disorders [56].

### Statistical analysis

#### Statistical methods for primary and secondary outcomes

The primary and secondary outcome variables will be analyzed using linear mixed models with “group” (placebo or SCFAs) as a between-subject factor and “visit” (pre- or postintervention) and, where appropriate, “timepoint” (measurement point within each visit) as within-subject factors. The group-by-visit and the group-by-visit-by-timepoint interaction effects constitute the principal effects of interest. If participants fail to exhibit an elevated cortisol stress response following stress induction, we will exclude their data from the analysis. To investigate the effect of the SCFAs intervention on treatment response, we will further control for BMI by including changes in BMI between visits 1, 2, and 3 in the linear mixed model as a continuous covariate. To investigate whether the changes in circulating SCFAs levels are mediated by the changes in the cortisol stress response to our stressor, we will pool all stress responders and enter the change in SCFA levels (post-intervention—pre-intervention) into the linear mixed model as a continuous covariate, including its interaction with visit, while omitting the group variable. The chosen significance level is alpha = 0.05. Follow-up planned contrasts will be conducted if there is a significant interaction effect and will be corrected for multiple testing comparisons using step-down Bonferroni (Holm) adjustment. Data of the intervention group will be further exploratively analyzed using linear mixed models with restrictive type AN and binge/purge type AN as “patient group,” investigating differences between the intervention of SCFAs between subtypes of patients with AN. In addition, we will enter the changes in questionnaires (baseline, post-intervention) into a linear mixed model as continuous covariates to investigate in an explorative way whether these changes (e.g., in AN severity, affect changes, subjective stress, etc.) are mediated by the changes in SCFAs levels and microbiota composition.

To examine restrictive food choice behavior using the FCT, we will use mixed effects logistic regression models on the binary choice data, where the participant's choice (selection of the trial-unique food item over the reference item) is the dependent variable, and z-scored healthiness and tastiness ratings are entered as independent variables. To assess self-control, we first categorize the trials as to whether they present a conflict between healthiness and tastiness ratings (food items rated tasty and unhealthy or non-tasty and healthy). In these trials, “self-control” is used when choosing healthy but non-tasty food or not choosing tasty but unhealthy food. Trials that elicit neutral responses are excluded. The proportion of trials with an opportunity for self-control and use versus nonuse of self-control will be modeled using mixed effects logistic regression.

#### Statistical methods for analyzing microbiome data

Pre-aliquoted fecal pellets are used for DNA extraction. Projected –omics analyses include metabolomic analysis, 16S/18S/ITS amplicon sequencing, transcriptomic, and metagenomic analysis. Statistical analyses on the microbiota will include estimation and comparison of microbiota diversity indices (observed richness, Shannon Index, Inverse Simpson Index, Pielou’s evenness index), samples' overall composition comparisons (using constrained ordination methods, such as principal coordinates and redundancy analysis), between-group analyses, and correlations with host metadata considering known confounders (such as weight loss and metabolic health) by using generalized linear models. Multiple testing corrections (Benjamini–Hochberg FDR) will be performed where applicable.

### Data management and monitoring

We will store data according to the ‘Research Data Management’ policy of KU Leuven. Research data will be collected and stored in an electronic case report form (eCRF) in the secure encrypted KU Leuven webserver. All data will be pseudonymized, with a key, only accessible to the primary researchers. Data gathered outside the eCRF will be collected under the pseudonym and later added to the eCRF. Investigators will promptly record any adverse events reported by the participant or observed by trial staff in the eCRF after detection. There are no plans for carrying out interim analyses. The UZ Leuven Clinical Trial Center assessed the clinical trial's risk and categorized it as having ‘comparable risk.’ This means that the potential risks associated with the study procedures and protocol design are deemed comparable to those of standard medical care. As a result, the trial's Sponsor decided that monitoring activities are not necessary. However, the Sponsor and the research ethics committee retain the right to audit and request source data from the trial at any time. The Sponsor or the principal investigator may terminate the trial prematurely at any time.

### Ethical aspects

The study is approved by the Medical Ethics Committee at the University Hospital Leuven in Belgium (reference S66404) and will be conducted in accordance with the Declaration of Helsinki. Prior to their admission into the research study, all participants must give their informed consent in a written format. Participants have the right to withdraw from the trial whenever they wish without incurring any disadvantages.

## Discussion

This protocol proposes a randomized control trial (RCT) using colon-delivery capsules (CDCs) to examine the effects of gut microbiota-affiliated metabolites, specifically SCFAs, on the stress response, eating behavior, and nutritional state in malnourished patients with AN. We focus on SCFAs for three primary reasons: First, preclinical data have evidenced the potential of SCFAs to modulate gut-brain interactions. Second, emerging research in healthy individuals has indicated that SCFAs can influence psychophysiological functions, including the stress response. Third, observed alterations in the gut microbiota composition of individuals with AN suggest a potential dysregulation in the production or utilization of SCFAs within this population. The employment of an RCT design paired with the use of CDCs allows us to examine the role of SCFAs in a mechanistic way. By administering SCFAs via CDCs, we ensure their direct delivery to the colon—their natural site of production—thereby enhancing the mechanistic insight to be gained from the study while maintaining its ecological validity.

Examining individuals with AN presents a distinct chance to explore the intricate interplay among the gut, brain, and behavior in psychiatric disorders, as eating disorders are most closely tied to nutrition and gut microbiota. Considering this, targeting the gut microbiota as part of AN treatment in the future could potentially be valuable in modulating the stress response, food choice, appetite, and neuropsychological functioning, among other outcomes.

## Data Availability

The datasets generated and/or analyzed during the current study are available from the corresponding author upon reasonable request.

## Abbreviations

ACTH: Adrenocorticotropin hormone
AN: Anorexia nervosa
BMI: Body mass index
CDCs: Colon-delivery capsules
DASS: Depression, anxiety, stress scales
DEBQ: Dutch eating behavior questionnaire
DSM-5: Diagnostic and statistical manual of mental disorders, 5th edition
eCRF: Electronic case report form
FCT: Food choice task
GLIMMPSE: General linear multivariate model power and sample size software
GSRS: Gastrointestinal symptom rating scale
HPA axis: Hypothalamus–pituitary–adrenal axis
IV: Intravenous
PANAS: Positive and negative affect schedule
PASA: Primary appraisal and secondary appraisal scale
PFS: Power of food scale
PSS: Perceived stress scale
RCT: Randomized controlled trial
sAA: Salivary alpha-amylase
SAM: Sympatho-adrenal medullary pathway
SCFAs: Short-chain fatty acids
TSST: Trier social stress test
UPC Z.Org: University Psychiatric Hospital Leuven
